# Monitoring Symptoms of COVID-19: Review of Mobile Apps

**DOI:** 10.2196/36065

**Published:** 2022-06-01

**Authors:** Suzanna Schmeelk, Alison Davis, Qiaozheng Li, Caroline Shippey, Michelle Utah, Annie Myers, Meghan Reading Turchioe, Ruth Masterson Creber

**Affiliations:** 1 Weill Cornell Medicine New York, NY United States; 2 St John's University Queens, NY United States; 3 Montefiore Medical Center Bronx, NY United States; 4 Memorial Sloan Kettering Cancer Center New York, NY United States

**Keywords:** COVID-19, mobile apps, mobile health, mHealth, symptom assessment, symptom tracking, public health, mobile health application, surveillance, digital surveillance, monitoring system, digital health

## Abstract

**Background:**

Mobile health (mHealth) apps have facilitated symptom monitoring of COVID-19 symptoms globally and have been used to share data with health care professionals and support disease prediction, prevention, management, diagnostics, and improvements in treatments and patient education.

**Objective:**

The aim of this review is to evaluate the quality and functionality of COVID-19 mHealth apps that support tracking acute and long-term symptoms of COVID-19.

**Methods:**

We systematically reviewed commercially available mHealth apps for COVID-19 symptom monitoring by searching Google Play and Apple iTunes using search terms such as “COVID-19,” “Coronavirus,” and “COVID-19 and symptoms.” All apps underwent three rounds of screening. The final apps were independently assessed using the Mobile Application Rating Scale (MARS), an informatics functionality scoring system, and the Center for Disease Control and World Health Organization symptom guidelines. The MARS is a 19-item standardized tool to evaluate the quality of mHealth apps on engagement, functionality, aesthetics, and information quality. Functionality was quantified across the following criteria: inform, instruct, record (collect, share, evaluate, and intervene), display, guide, remind or alert, and communicate. Interrater reliability between the reviewers was calculated.

**Results:**

A total of 1017 mobile apps were reviewed, and 20 (2%) met the inclusion criteria. The majority of the 20 included apps (n=18, 90%) were designed to track acute COVID-19 symptoms, and only 2 (10%) addressed long-term symptoms. Overall, the apps scored high on quality, with an overall MARS rating of 3.89 out of 5, and the highest domain score for functionality (4.2). The most common functionality among all apps was the instruct function (n=19, 95%). The most common symptoms included in the apps for tracking were fever and dry cough (n=18, 90%), aches and pains (n=17, 85%), difficulty breathing (n=17, 85%), tiredness, sore throat, headache, loss of taste or smell (n=16, 80%), and diarrhea (n=15, 75%). Only 2 (10%) apps specifically tracked long-term symptoms of COVID-19. The top 4 rated apps overall were state-specific apps developed and deployed for public use.

**Conclusions:**

Overall, mHealth apps designed to monitor symptoms of COVID-19 were of high quality, but the majority of apps focused almost exclusively on acute symptoms. Future apps should also incorporate monitoring long-term symptoms of COVID-19 and evidence-based educational materials; they should also include a feature that would allow patients to communicate their symptoms to specific caregivers or their own health care team. App developers should also follow updated technical and clinical guidelines from the Center for Disease Control and the World Health Organization.

## Introduction

Monitoring and tracking acute short-term symptoms are important to help identify the combination of symptoms that occur in individuals with COVID-19 for personal and public health purposes [1]. Information gathered from public symptom tracking can also help guide recommendations for self-isolation and testing and help prevent further spread of the virus [2]. The Center for Disease Control (CDC) recommends daily monitoring for symptoms of COVID-19 illness to reduce transmission risk [1], and to holistically understand its full impact.

In addition to short-term symptoms, there is a growing awareness of the long-term symptoms of COVID-19 including fatigue or loss of taste and smell, as well as multiorgan effects on the heart, lungs, renal function, and cognitive functions [3]. The population prevalence of long COVID is unknown but is estimated to affect between 1 in 5 people (symptoms beyond 5 weeks) and 1 in 10 people (symptoms 12+ weeks) [4] who have been infected with COVID-19. Improved understanding of long-COVID symptoms can help health care professionals recognize the most common long-term impacts and add urgency to the public health messaging focused on the importance of COVID-19 vaccination efforts [5].

Since the start of the pandemic, mobile health (mHealth) [6] apps have been leveraged in several ways to control the spread of COVID-19 [7]. These mHealth apps can be used to monitor symptoms, share data with providers, and support disease prediction, prevention, management, diagnostics, and improvements in treatments and patient education, ultimately giving patients more control [8]. The majority of adults in the United States have access to a smartphone (>85%) [9]; thus, mHealth apps are poised to provide scalable and cost-effective delivery of health care at the point of need for patients with COVID-19. The incorporation of pertinent epidemiological and geographic data on the presence of transmittable diseases in a region allows the tracing of cases, which can be used as a successful tool to control the spread of the infection [10]. The mHealth apps can also help solve several COVID-19–related challenges by increasing the reach of reliable information to both patients and health care professionals. Additionally, mobile apps can assist in tracking physical and mental health symptoms, support home monitoring, and reduce the burden of hospitals [7].

The aim of this review was to evaluate the quality and functionality of mHealth apps that support tracking acute and long-term symptoms of COVID-19. Our research focuses on the CDC [1,3,11] and World Health Organization (WHO) [12] symptom guidelines tracked by the apps as medical understandings continue to develop over time. Our analysis can support health care professionals by identifying appropriate mHealth apps for patients regarding COVID-19 acute and long-term symptoms. This review also identified key areas in the quality, functionality, and content of existing COVID-19 apps that can be improved in current and future related app development.

## Methods

### Systematic Search Criteria and Selection

In June 2021, we systematically searched 2 major app stores: the Apple App Store and Android Google Play Store. In the 2 app stores, search terms included “COVID-19,” “Coronavirus,” “COVID-19 and Symptoms,” “Coronavirus and Symptoms,” “COVID-19 and Symptom Monitoring,” and “Coronavirus and Symptom Monitoring” to identify relevant apps. Following subsequent searches of “COVID-19” and “Coronavirus,” the authors found that the results were the same. The terms “COVID-19,” “Coronavirus,” “COVID-19 and Symptoms,” and “COVID-19 and Symptom Monitoring” were then collapsed to streamline the search.

All apps underwent three rounds of screening. During the first round, the title and screenshots of the apps were used to exclude those that were exclusively for contact tracing (without symptom tracking functionality), were not available in English, required an institution-specific login, were clinical guidelines for clinicians, were games, or provided general or other COVID-19–related information. During the second round of review, screenshots and descriptions of the apps were reviewed to exclude apps that were too general, only had a singular function (eg, only track and trace), or were not relevant to tracking COVID-19 symptoms. During the third round of review, the final apps were downloaded, and apps requiring institutional credentialing were excluded.

### Evaluation Measures or Rating Tools

All apps were evaluated using the Mobile Application Rating Scale (MARS) [13,14], IQVIA for Healthcare Informatics [15] functionality scoring system, and specific COVID-19 symptoms according to the CDC [1,3,11] and WHO COVID-19 health topic guidelines [12]. Both the MARS and IQVIA functionality scores were used for this review. The MARS functionality score focuses on performance, ease of use, navigation, and gestural design of the app [13]. The 7 IQVIA functionality scores focus on the scope of functions, including informing, instructing, recording, displaying, guiding, reminding, and communicating information [14].

The MARS [13] is a widely used, multidimensional tool to evaluate the quality of mobile health apps and was developed based on semantic analysis and a combination of relevant literature. The MARS was used to rate app quality and includes four sections: classification, quality, satisfaction, and a modifiable app-specific section. The classification section provides descriptive information about the apps. The objective app quality section includes 19 items divided into four scales: engagement, functionality, aesthetics, and information quality. MARS items are scored using a 5-point Likert scale (1=inadequate, 2=poor, 3=acceptable, 4=good, and 5=excellent) [13]. The final MARS scores include a total mean score for the engagement, functionality, aesthetics, and information subscales. In addition, the MARS also includes a subjective quality score and an app-specific subscale that assesses perceived effect on the user’s knowledge, attitudes, and intentions to change, as well as the likelihood of changing the identified targeted behaviors. The functionality domain measures whether an app is easy to learn, encourages seamless navigation, supports a logical flow, and examines the overall app gestural design. The engagement score includes evaluation of whether the app is fun, interesting, customizable, and interactive (eg, sends alerts, messages, reminders, and feedback, and enables sharing), and is well targeted to the intended audience.

The IQVIA functionality score (formerly termed the IMS functionally score) is based on 7 functionality criteria and 4 functional subcategories as described in detail in the IQVIA Institute for Healthcare Informatics report [14]. IQVIA is not an acronym; the name represents a merger between two companies, IMS Health and Quintiles. The apps were evaluated on the 7 functionality criteria of inform, instruct, record, display, guide, remind or alert, and communicate. The record functionality criteria were further scored on 4 subcategories: collect (ability to enter or store data on individual phone), share (ability to transmit data), evaluate (ability to evaluate health data by caregiver or health care entity), and intervene (ability to alert in response to collected data or propose behavioral intervention).

The specific symptoms tracked in each app were evaluated against acute and long-term symptoms of COVID-19 as reported by the CDC [1] and the WHO [12] as of June 2021. Acute COVID-19 symptoms included fever, dry cough, tiredness, aches and pains, sore throat, diarrhea, conjunctivitis, headache, loss of taste or smell, skin rash or discoloration of fingers or toes, difficulty breathing or shortness of breath, chest pain or pressure, and loss of speech or movement. Long-COVID-19 symptoms, defined as more than 4 weeks from the initial infection [3], included tiredness or fatigue, difficulty thinking or concentrating (“brain fog”), headache, loss of smell or taste, dizziness while standing, heart palpitations, chest pain, difficulty breathing or shortness of breath, cough, joint or muscle pain, depression or anxiety, fever, and symptoms that worsen after physical or mental activities. Symptoms that are consistent with both short-term and long-term COVID-19 (eg, cough), were evaluated based on how data entry was described in the app and the current version of the app.

### Data Extraction and Data Analysis

We created a Google data extraction form consisting of questions from (1) the MARS questionnaire [13], (2) IQVIA functionality guidelines [14], and (3) the CDC [1] and WHO [12] COVID-19 symptoms. A total of 5 reviewers independently evaluated the 4 randomly selected apps using the data extraction form to assess interrater reliability, which was acceptable (0.75-0.83). Domains with low agreement between reviewers (<0.7) were discussed until consensus was reached, apps were rereviewed, and interrater reliability was recalculated. The remaining apps were independently evaluated by 2 reviewers. Mean MARS scores for each domain, MARS total scores, and IQVIA functionality scores are reported.

## Results

**Figure 1:**
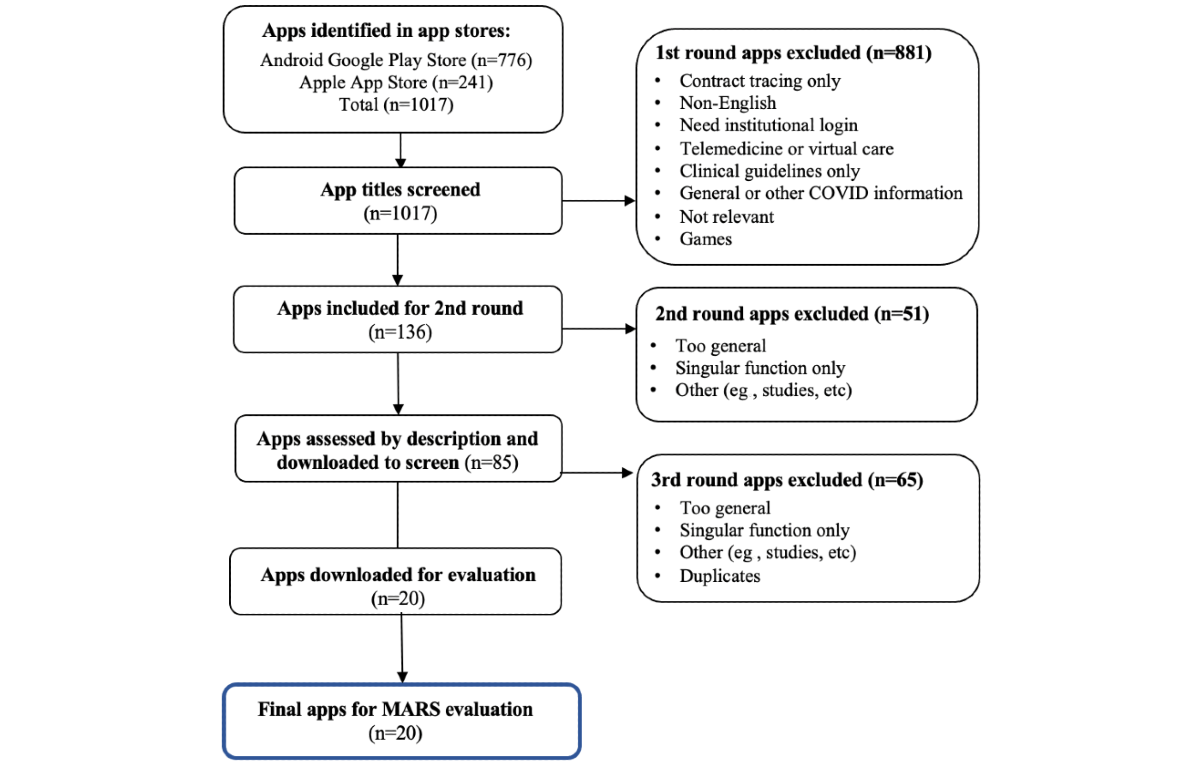
PRISMA (Preferred Reporting Items for Systematic Reviews and Meta-Analyses) flowchart of the screening process. MARS: Mobile Application Rating Scale.

The Android Google Play and Apple App Store searches identified 1017 potentially relevant apps, of which 20 (2%) met our final inclusion criteria. The search strategy (Figure 1) shows the number of apps included and excluded in each round of review. In total, 50% (n=509) of the apps had a government affiliation, 10% (n=102), were affiliated with a university and the rest (n=406, 40%) were developed by private companies (Table 1).

**Table 1:** Descriptive characteristics of the apps included.

App name	Star rating	N ratings	Current version	Last update	Affiliation	Geographic location–dependent?	Privacy policy
Apollo	4.8	16	1.2.8	2020	Commercial^a^	No	Yes
Beebe Covid-19 Screening Tool	3	2	N/A^b^	N/A	Commercial^a^	No	Yes
Geohealthapp Covid19 Tracker	3.6	8	1.2	2020	Commercial^a^	No	Yes
Healthy Together - Covid-19	4.9	71,219	1.5.8	2021	Commercial^a^	No	Yes
HowWefeel	4.8	54,879	1.13.3	2020	Commercial^a^	No	Yes
Patientsphere Cv	2.8	9	1.1	2020	Commercial^a^	No	Yes
Apple Covid-19	4.2	2782	5	2020	Commercial^a^	No	Yes
Bc Covid-19 Support	3.4	7	1.41	2021	Government	Yes	Yes
Care 19 Diary	3.8	591	3.6	2020	Government	Yes	Yes
Covid Alert DE	4.2	30	1.2.2	2020	Government	Yes	Yes
Covid Alert NJ	3.7	301	1.1.4	2021	Government	Yes	Yes
Covid Alert NY	4.6	544	1.1.7	2021	Government	Yes	Yes
Covid Alert Pennsylvania	4.1	301	2.0.0	2021	Government	Yes	Yes
Covid Coach	4.8	722	1.6	2021	Government	No	Yes
Covid Trace Nevada	2.8	172	1.2.16	2021	Government	Yes	Yes
Crush Covid RI	3.6	195	3	2020	Government	Yes	Yes
Soco Covid-19 Check	2.3	50	1.1.2	2020	Government	Yes	Yes
Check Covid	4.5	74	3	2021	University	No	Yes
My Covid-19 Tracker	3.8	5	1.2.2	2021	University	No	No
Canada Covid-19	4.4	16	5.17	2021	Commercial^a^	Yes	Yes

^a^For-profit.

^b^N/A: not applicable.

### Mobile Application Rating Scale

The average overall MARS rating for the 20 apps was 3.89 with the functionality domain having the highest score (4.20) and the engagement score having the lowest average score (3.52)*.* The top 4 rated apps overall were state-specific apps developed and deployed for public use in New York, Pennsylvania, Rhode Island, and New Jersey (Table 2), between July and October 2020*.*

**Table 2:** Mobile Application Rating Scale (MARS) quality scores.

App name	Engagement	Functionality	Aesthetics	Information	Overall score
Covid Alert NY	4.30	4.50	4.83	4.00	4.41
Covid Alert Pennsylvania	4.00	4.13	4.83	4.00	4.24
Crush Covid RI	4.05	4.44	4.42	4.04	4.23
Covid Alert NJ	4.00	4.50	4.50	3.86	4.21
HowWeFeel	3.90	4.55	4.47	3.80	4.18
Covid Coach	3.90	4.63	4.67	3.43	4.16
Healthy Together: Covid-19	3.92	4.40	4.47	3.74	4.13
Check Covid	4.00	4.63	4.33	3.29	4.06
Canada Covid-19	3.60	4.50	4.33	3.71	4.04
Covid Alert DE	3.60	4.63	4.17	3.64	4.01
Apple Covid-19	3.60	4.33	4.22	3.74	3.97
SoCo Covid-19 Check	3.60	4.25	4.00	3.79	3.91
My Covid-19 Tracker	3.30	3.88	4.00	3.64	3.70
Apollo Covid-19	2.95	4.00	4.17	3.64	3.69
Beebe Covid-19 Screening	3.10	4.00	3.67	3.64	3.60
Care19 Diary	2.90	3.50	4.00	3.71	3.53
Patientsphere CV	3.10	4.00	3.50	3.43	3.51
BC Covid-19 Support	3.12	3.85	3.53	3.43	3.48
Covid Trace Nevada	3.00	3.58	3.67	3.57	3.46
Geohealthapp Covid19 Tracker	2.40	3.75	3.83	2.79	3.19
Average score	3.52	4.20	4.18	3.64	3.89

### IQVIA Functionality

**Figure 2:**
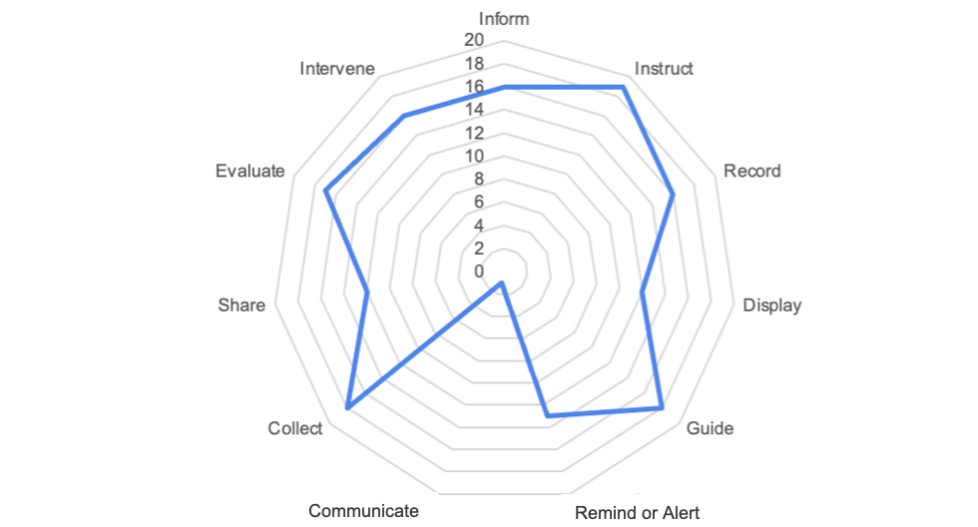
Functionality scores based on IQVIA for Healthcare Informatics.

Overall, the “instruct” function was the most common among all apps (n=19, 95%) (Figure 2). In many of the apps, the instruct function aided in directing users on next steps based on the symptoms that were recorded. The “Beebe COVID-19” screening app was the only app that had the ability to communicate between patients and health care providers. There were no apps that had all 11 functionalities. Another domain that the majority of the apps had was the collect function (n=18, 90%). These apps collected patients’ daily COVID-19 symptoms and collected some demographic information (n=18, 90%) including: age, name, date of birth, ethnicity, and geographical location.

### COVID-19 WHO and CDC Guidelines

The acute COVID-19 symptoms tracked in each app (Table 3), and long-term symptoms (Table 4) were evaluated against the recommendations provided by the CDC [1] and the WHO [12]. The majority of apps focused on acute COVID-19 symptoms (n=18) with the most common acute symptoms including fever and dry cough (n=18, 90%), aches and pains (n=17, 85%), difficulty breathing (n=17, 85%), tiredness, sore throat, headache, loss of taste or smell (n=16, 80%), and diarrhea (n=15, 75%).

**Table 3:** COVID-19 acute symptoms.

App name	Fever	Dry cough	Tiredness	Aches and pains	Sore throat	Diarrhea	Conjunctivitis	Headache	Loss of taste or smell	Skin rash	Difficulty breathing or shortness of breath	Chest pain or pressure
Apollo	✓^a^	✓	✓	✓	✓	✓	—^b^	✓	✓	—	✓	—
Apple Covid-19	✓	✓	✓	✓	✓	✓	—	✓	✓	—	✓	✓
BC COVID-19 Support	✓	✓	✓	✓	✓	—	—	✓	✓	✓	✓	✓
Beebe COVID-19 Screening Tool	✓	✓	—	✓	✓	✓	—	✓	✓	✓	✓	—
Canada COVID-19	✓	✓	✓	✓	✓	✓	—	✓	✓	✓	✓	—
Care19 Diary	✓	✓	✓	—	—	—	—	—	—	—	✓	—
Check Covid	✓	✓	✓	✓	✓	✓	—	✓	✓	—	✓	✓
COVID Alert DE	✓	✓	✓	✓	✓	✓	—	✓	✓	—	✓	—
COVID Alert NJ	✓	✓	✓	✓	✓	✓	—	✓	✓	—	✓	✓
COVID Alert NY	✓	✓	✓	✓	✓	✓	—	✓	✓	—	✓	—
COVID Alert Pennsylvania	✓	✓	✓	✓	✓	✓	—	✓	✓	—	✓	—
COVID Trace Nevada	✓	✓	✓	✓	✓	✓	—	✓	✓	—	✓	—
Crush Covid RI	✓	✓	✓	✓	✓	✓	—	✓	✓	—	✓	—
Healthy Together	✓	✓	✓	✓	✓	✓	—	✓	✓	—	✓	—
HowWeFeel	✓	✓	✓	✓	✓	✓	—	✓	✓	—	✓	✓
My COVID-19 Tracker	✓	✓	✓	✓	✓	✓	✓	✓	✓	✓	✓	✓
PatientSphere	✓	✓	✓	✓	✓	—	—	—	—	—	—	—
SoCo COVID-19 Check	✓	✓	—	✓	—	✓	—	✓	✓	—	✓	—

^a^✓ implies that the symptom is measured in the app.

^b^— implies that the symptoms are not measured in the app.

**Table 4:** COVID-19 long-term symptoms.

App name	Dry cough	Tiredness	Headache	Loss of taste or smell	Symptoms that worsen after physical or mental activities	Difficulty breathing or shortness of breath	Chest pain or pressure	Depression or anxiety
BC COVID-19 Support	✓^a^	✓	✓	✓	✓	✓	✓	—^b^
HowWeFeel	—	—	—	—	—	—	—	✓

^a^✓ implies that the symptoms are measured in the app.

^b^— implies that the symptoms are not measured in the app.

## Discussion

### Principal Findings

The aim of this review was to evaluate COVID-19 mHealth apps that support tracking acute and long-term symptoms of COVID-19. The majority of the apps reviewed focused on acute symptoms, including fever and dry cough, aches and pains, difficulty breathing, tiredness, sore throat, headache, loss of taste or smell, and diarrhea. Few apps measured long-term symptoms of COVID-19. Overall, the apps scored high on quality, and the most common functionality was providing the user with instructions on what to do in response to symptoms.

Previous reviews of mHealth apps for COVID-19 were published by Alanzi [16] and Davalbhakta [17]. The review published by Alanzi [16] reviewed apps from 7 countries (Saudi Arabia, India, Singapore, Australia, Italy, United Kingdom, and the United States). These apps were reviewed on functionality (eg, purpose, services offered, and networking technologies) and effectiveness (eg, learnability, communication strategies, and design). There were no overlapping apps between the review published by Alanzi [16] and this review. The review by Davalbhakta and colleagues [17] was consistent methodologically by using the MARS to review the apps, in addition to adding more functionalities specific to COVID-19 (eg, individual tracking, contact tracing, and health care worker training). The review by Davalbhakta [17] and this review shared 6 common apps (Apollo, PatientSphere, Apple COVID-19, Bc COVID-19 Support, Check COVID, and Canada COVID-19). Other reviews did not evaluate short- and long-term symptom monitoring according to the CDC [1] or WHO COVID-19 health topic guidelines [12].

The highest quality apps were developed by individual states within the United States to support acute COVID-19 symptom tracking. Overall, the state-specific apps had the most up-to-date and accurate information, which could be attributed to collaborative efforts between local, county, or state departments of health [18-23]. The state-specific apps all listed most of the common symptoms and provided information about local COVID-19 case counts and developments in research. Strengths of the state-specific apps included long-term storage of the symptom evaluations, high-quality aesthetics, comprehensive symptom lists, and evidence-based information that was aligned with CDC guidelines [1]. While the contact tracing functions were likely most useful to in-state residents, the symptom tracking functionalities were available for anyone regardless of their home state.

At the time of app development and deployment, less was known about long-term symptoms of COVID-19 [24]. Only 2 (10%) apps specifically tracked long-term symptoms of COVID-19 (Table 4), though the symptoms of long COVID-19 are increasingly concerning [25-27]. As such, mHealth apps that track long COVID-19 symptoms could play a significant role in helping to manage them more efficiently and gather additional data about how this disease is affecting patients over a long period of time [25-27]. The authors suggest that app developers should follow updated technical and clinical guidelines from the CDC and the WHO to ensure consistency and efficacy of long-term symptom monitoring. Future research should focus on expanding these apps to support patients with long-term symptoms [3] and providing educational materials about the disease.

The largest gap in app functionality was in the communication feature, which could be further developed to allow patients to communicate their symptoms to chosen family members for supportive care or their own health care professionals. These algorithms are useful for providing basic information about what to do in response to the symptoms and quarantine guidance. However, many patients, especially those with complex care needs, require additional support and communication with their health team to manage long-term symptoms of COVID-19. There are specific legal and regulatory issues around data sharing that are likely the reasons why many apps do not actively support more data-sharing features. Additional areas of future development include expansion into pediatric populations and long-term symptom tracking integrated into mHealth apps. Lastly, these apps should be evaluated for usability and inclusivity characteristics, including non-English languages.

### Limitations

A limitation of this review is that it provides a snapshot of the app landscape at a specific point in time when the app stores have continued to rapidly evolve. Many of the apps are also going on and off the market based on the introduction of novel variants. To mitigate the limitation of subjectivity of reviewing apps, we applied a rigorous multistep methodology including using the MARS [13,28,29] and IQVIA for Healthcare Informatics [15] functionality scoring system and mapping all the apps to the CDC and WHO COVID-19 health topic guidelines for symptom monitoring.

### Conclusion

In general, mHealth apps for tracking COVID-19 symptoms offer a potential solution to help people identify and track virus symptoms. Overall, the mHealth apps had high quality, considering the expedited needs during the COVID-19 pandemic. Future apps should also incorporate monitoring long-term symptoms of COVID-19 and include a communication feature that could allow patients to communicate their symptoms to specific caregivers or their own health care team.

## Abbreviations

CDC: Center for Disease Control
MARS: Mobile Application Rating Scale
mHealth: mobile health
WHO: World Health Organization
